# Crossing Language Barriers: Treating Chronic Low Back Pain in Panama With the Fascial Distortion Model

**DOI:** 10.7759/cureus.106198

**Published:** 2026-03-31

**Authors:** Julie L Herek, Ashley Ban, Quinton Nace, Jacob Traverse, Nathan L Tebben, Carol Penn

**Affiliations:** 1 Osteopathic Medicine, Rocky Vista University Montana College of Osteopathic Medicine, Billings, USA; 2 Family Medicine, Rocky Vista University Montana College of Osteopathic Medicine, Billings, USA

**Keywords:** chronic low back pain (clbp), fascial distortion model, global health care, herniated trigger point, nonverbal communication, osteopathic manipulative treatment (omt), trigger band

## Abstract

The fascial distortion model (FDM) is an anatomical perspective and treatment technique within the discipline of osteopathic manipulative treatment (OMT). The FDM is unique in that it elucidates a set of common gestures and body language for each of the six recognized FDM distortions. Fascia not only surrounds structures but also links them, transmitting mechanical forces throughout the body in a continuous three-dimensional network. Dysfunction within this system, whether local or distant, can contribute to distinct patterns of body morphology. Although fascial anatomy provides a framework for identifying chains of injury, variations in palpated tension may point toward different underlying pathologies and guide clinical interpretation. Thus, a skilled palpation can detect areas of functional conflict and pain without verbalization. This case study explores the application of FDM in treating chronic low back pain in a Panamanian agricultural worker, highlighting its utility across language barriers. B.P. presented with chronic low back pain, limited range of motion (ROM), and hypertonicity of the lumbar paraspinal muscles. Lumbar active ROM was assessed using visual estimation before, during, and after treatment. B.P.’s nonverbal gestures indicated two trigger bands and two herniated trigger points, which were treated using the appropriate FDM techniques. Post-treatment evaluations revealed improvement in functional mobility and complete resolution of pain. B.P. demonstrated increased lumbar ROM and reported relief without requiring extensive verbal communication. This case report demonstrates the effectiveness of using the FDM to treat chronic low back pain, while giving credence to the universal nature of the body language described by the model. In B.P.’s case, FDM treatments facilitated a successful and culturally competent intervention that improved both pain and function, reinforcing the value of this treatment modality in global and resource-limited contexts.

## Introduction

The fascial distortion model (FDM), introduced in 1991 by Stephen Typaldos, D.O., is an anatomical perspective through which musculoskeletal, medical, and some neurological conditions are seen as pathological disruptions of the underlying fascia [1]. FDM focuses on mechanically correcting specific fascial distortions rather than targeting muscle relaxation or joint mobilization like traditional osteopathic techniques. Dr. Stephen Typaldos, a family physician, developed the model after observing recurring patient gestures that corresponded with fascial pathologies. Over time, he refined these clinical observations into six distinct fascial distortions, each associated with its own set of pathognomonic gestures and body language. The FDM body language has been shown to have higher inter-rater reliability, making it a helpful tool for novice and experienced practitioners alike [2]. The high reliability of this “universal body language” has many potential applications, including ease of diagnosis and assessment when language barriers pose a challenge. This provides physicians with a well-rounded approach to assist a wide variety of patients while maintaining a balanced perspective regarding treatment.

## Case presentation

B.P. was a 46-year-old Panamanian male who presented to the clinic for an initial evaluation of chronic lower back pain secondary to intensive physical labor. His pain has been present for the past eight years and recurs when he is physically active for agricultural work. He does not have secure access to over-the-counter anti-inflammatory or pain medications, and denied any prior tests or treatment regarding his low back pain. B.P. rated his pain as 3/10 on the numeric pain scale. This pain was debilitating and interfered with his ability to perform activities of daily living.

**Figure 1:**
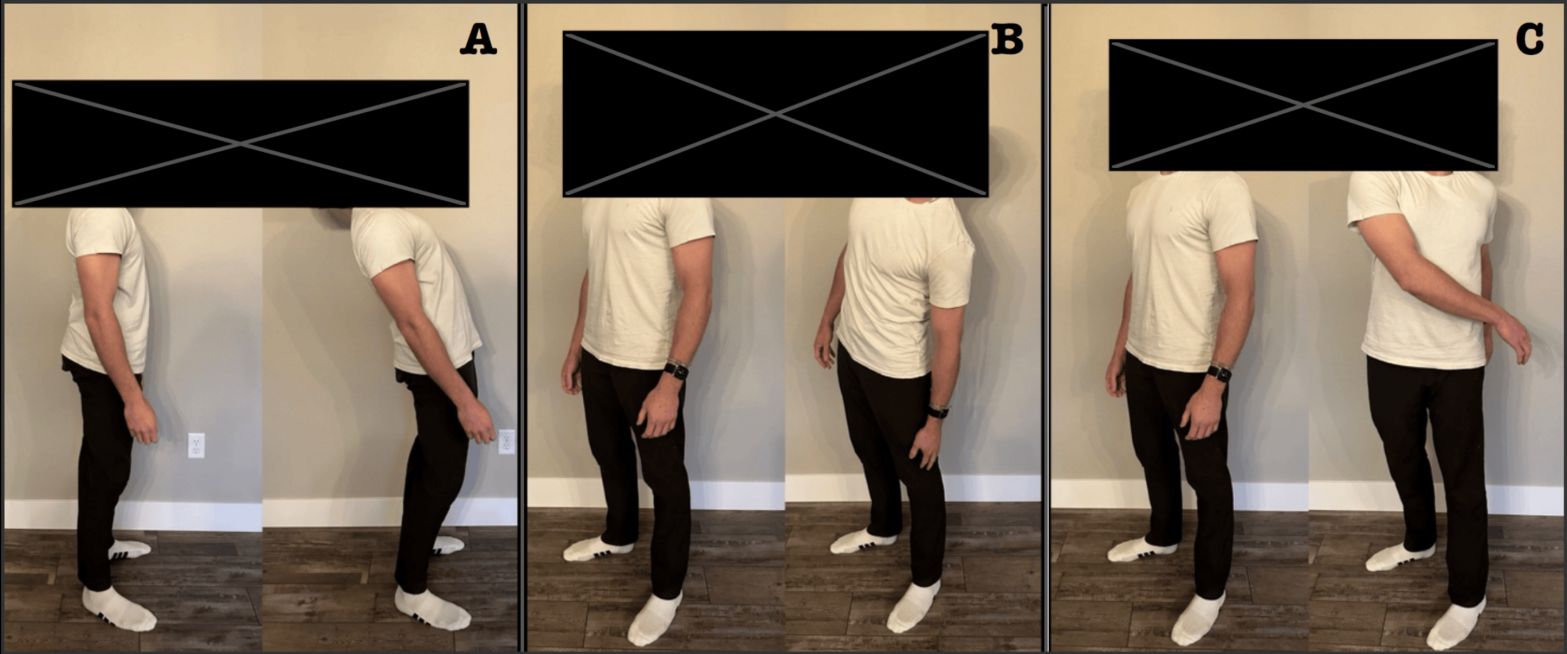
Range of motion testing in extension (A), side bending (B), and rotation (C).

Initial evaluation included a musculoskeletal examination focused on the lumbar spine and lower extremities. In cases of lower back pain of fascial origin, assessment of upper limb mobility is important, as the fascia of the trapezius and latissimus dorsi is functionally continuous with the thoracolumbar and spinal fascia. However, this evaluation was not performed during the visit. Physical exam revealed hypertonicity of the left lumbar paraspinal muscles, and range of motion (ROM) testing revealed pain with lumbar extension, right side bending, and left rotation (Figure 1).

The lower extremities demonstrated 5/5 strength bilaterally with mild crepitus in the left knee. After a focused examination, B.P. was diagnosed with a repetitive strain injury of the lumbar spine.

**Figure 2:**
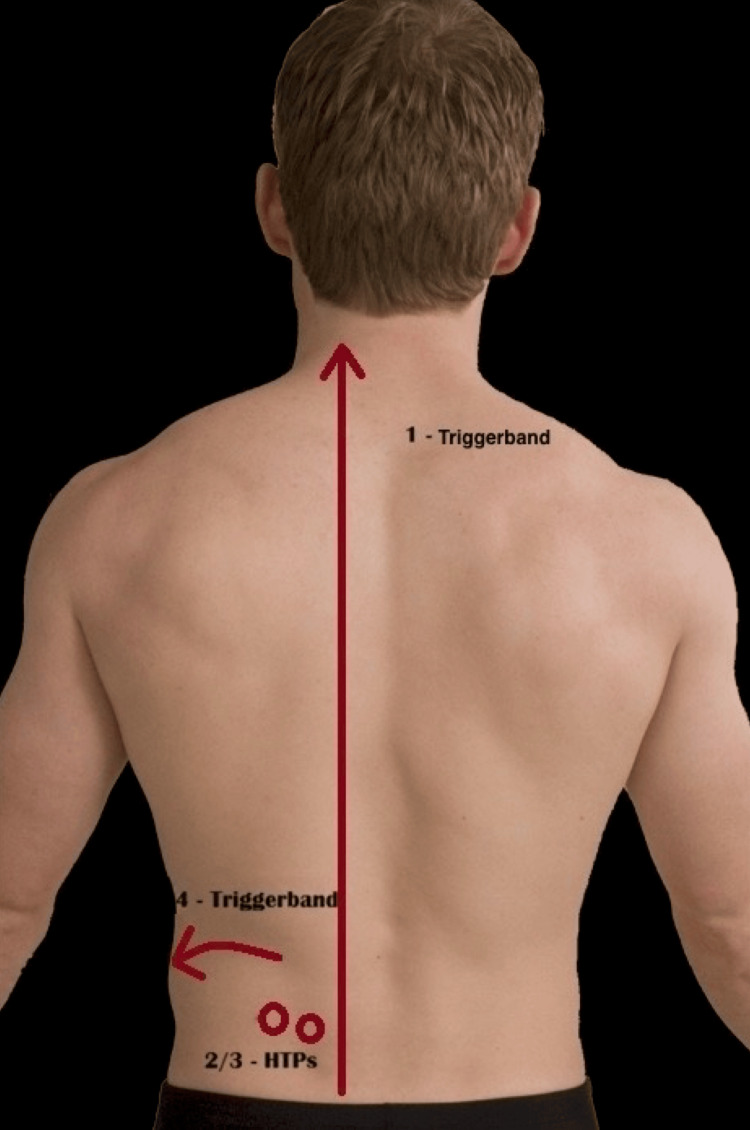
Surface marking of areas of diagnosis and treatment. HTPs: herniated trigger points.

After assessing B.P.’s indications for osteopathic manipulative medicine and evaluating for any contraindications, he agreed to participate in treatment. When asked to show where the pain was, he traced a line with his thumb following his left erector spinae muscles, 4-6 cm lateral to his spine (trigger band (TB)), as well as two specific areas along the line that he found particularly painful, herniated trigger points (HTPs) (Figure 2). Following principles of FDM, high pressure was delivered with the thumb, drawing a line from the iliac crest, passing through the indicated line of pain, and continuing up to the base of the cranium, remaining about 4-6 cm left of the spine (Video 1). Video 1 illustrates the initial phase of this intervention; however, a more comprehensive recording demonstrating the full treatment sequence, with the inclusion of subtitles, is necessary to enhance instructional value. High pressure with the thumb was delivered to the two HTPs. ROM was retested, revealing significant improvement in accessible range, but B.P. indicated another TB that began ~4 cm superior to the iliac crest and ~6 cm lateral to the lumbar spine, which extended laterally over the quadratus lumborum on the left. High pressure was delivered with the thumb, drawing a line from just lateral to the spinous process at the level of the indicated pain, through the TB, and stopped on the mid-axillary line. ROM was retested, revealing further improvement in accessible range and resolution of pain. The patient tolerated the treatment well. Changes were immediate; however, no follow-up occurred, so long-term outcomes could not be assessed. No adverse effects were noted during or immediately after treatment.

**Video 1:** Beginning of the treatment for a trigger band.

After osteopathic manipulative treatment (OMT), B.P. was prescribed oral ibuprofen and diclofenac cream, with appropriate patient education on when and how to use them to alleviate his low back pain.

## Discussion

Universal hand gestures have appeared throughout history in religious and artistic traditions, suggesting symbolic meaning across cultures. Cross-cultural studies have shown that while some gestures vary between societies, others, such as pointing, are consistently understood. Psychologist Paul Ekman’s work further demonstrated that basic emotions are universally expressed and recognized, reinforcing the importance of nonverbal communication [3,4]. FDM extends this principle into clinical medicine by decoding gestures as indicators of specific fascial pathologies (Table 1).

**Table 1:** Fascial distortions, their underlying pathology, and their associated body language. Source references: [1,5].

Distortion	Pathology	Associated body language
Trigger band (TB)	Twisted or wrinkled fascial banded fascia	Sweeping motion using one or more fingers in a line
Herniated trigger point (HTP)	Protrusion of fascia through a more superficial fascial plane	Pressing with multiple fingers or a thumb
Continuum distortion (CD)	Alteration at the transition zone of tissue types (most often muscle insertions)	Pointing with a single finger
Folding distortion (FD)	Three-dimensional alteration of fascial planes, often in a joint	Cupping or squeezing of the joint with the hand
Cylinder distortion (CyD)	Overlapping of cylindrical fascial coils	Repeatedly squeezing or sweeping the palm over the affected area
Tectonic fixation (TF)	Inability of the fascia to appropriately glide, becoming stuck	Attempts to forcefully move the joint

The therapeutic effects of the FDM are thought to arise from the correction of specific alterations in fascial structure and tension that disrupt normal force transmission and proprioceptive signaling. By applying targeted manual forces based on patient-reported pain patterns and gestures, FDM aims to restore fascial continuity, reduce nociceptive input, and normalize movement. This approach differs from traditional osteopathic techniques, which often emphasize muscle relaxation, joint mobilization, or reflex-mediated changes. FDM is directly focused on mechanically correcting discrete fascial distortions. In this case, the rapid improvement in pain and function following treatment supports a primarily mechanical and neuromyofascial mechanism. These findings suggest that FDM may offer a complementary approach for patients with functionally driven musculoskeletal pain, particularly in settings where rapid recovery is essential and follow-ups are limited. Further investigation is needed to better characterize its mechanisms and to compare its efficacy with established manual therapies.

In this case, B.P.’s nonverbal gestures, combined with palpatory findings, allowed for the identification and treatment of trigger bands and herniated trigger points without requiring detailed verbal explanation. These treatments resulted in immediate improvements in pain and function.

## Conclusions

This case suggests that FDM can effectively treat chronic low back pain while transcending cultural and linguistic barriers. In B.P.’s case, FDM treatments facilitated a successful and culturally competent intervention that improved both pain and function. The treatment results highlight the practicality of FDM in resource-limited settings where time, equipment, and communication tools may be constrained. Despite the outcomes of this case, additional research is necessary to validate these findings in broader populations. This case reinforces the value of FDM as an accessible and patient-centered modality that can enhance global health efforts, support cross-cultural care, and broaden the scope of OMT in diverse clinical environments.
